# An international primary care workforce: addressing the ethics beyond the red list

**DOI:** 10.3399/BJGP.2025.0243

**Published:** 2025-06-27

**Authors:** Laura Emery, Vincent Faseyosan, Sophie Park

**Affiliations:** School of Medicine and Population Health, University of Sheffield, Sheffield, UK; Doncaster and Bassetlaw Hospitals NHS Foundation Trust, Doncaster Royal Infirmary, Doncaster, UK; Nuffield Department of Primary Care Health Sciences, University of Oxford, Oxford, UK

In March 2025, the Nuffield Trust published a report^1^ that examined ‘Health in the UK after Brexit’. This highlighted the instability of the UK healthcare workforce following the end of free movement of labour in 2021. Nine per cent of UK doctors are now from countries on the World Health Organization ‘red list’. These countries have so few healthcare professionals that high-income nations are discouraged from recruiting their staff lest this further impact the availability of local health care.^2^ Wes Streeting, the UK’s Secretary of State for Health, has suggested that failure to train enough healthcare staff in the UK has left the NHS with an immoral dependence on staff from these nations.^3^ A key objective of the UK Government’s code of practice for recruitment of international health and social care workers^4^ is to prevent ‘active recruitment’ from red-listed countries. In response to the Nuffield report, WHO director Jim Campbell suggested that the UK is not breaching rules by proactive recruitment; rather, individuals are directly seeking employment in the UK.^5^

A systematic review examining the drivers of healthcare workers’ migration found that low income and personal security were key macro-level motivators for individuals to move away from their home country.^6^ Examples of such security issues can include experiences of persecution, risk of kidnapping, and personal attacks on healthcare staff *en route* to, or within, the workplace. On a meso-level, there is movement towards improved career prospects, better working conditions, and an increase in job satisfaction.^6^ Regardless of motivation, on arrival in the UK, international medical graduates (IMGs) currently find it harder to access training: only 16% of all non-UK graduate licensed doctors in 2023 had secured a training place.^7^ For those doctors in training, differential attainment in Annual Reviews of Competency Progression (ARCP)^8^ and postgraduate exams remains a significant issue.^9^

The UK continues to need more doctors as we deal with the complex challenges of population growth and the needs of an ageing population. Medical school places have been expanded to meet this demand, and the current NHS Long Term Workforce Plan promises a 50% increase in the number of GP specialty training places by 2032, although at the time of publication this is currently under active review.^10^ Despite reports of a 26% increase in the number of GP trainees, overall the number of fully qualified full-time equivalent GPs has fallen by 3.8% since 2015.^11^

Nationally, there is a 23% increase in the number of doctors joining the General Medical Council register,^12^ driven by IMGs who account for two-thirds of joiners. In 2023, 52% of GP trainees were non-UK graduates. It would be naïve however to assume that this is novel; since the 1930s the UK healthcare system has benefited from migrants to expand the workforce.^13^ As a nation, and perhaps especially since Brexit, we have a preoccupation with ‘home-grown’ healthcare professionals. This is problematic from a workforce point of view considering our lead time for a fully qualified GP is a minimum of 10 years, therefore resulting in a significant lag between demand and supply. This also fails to acknowledge the importance of embedding international examples and experience into our curricula so that we can benefit from a wider world view of health care rather than simply what is relevant to the UK and the NHS.

The Nuffield report suggests that, because it is now more difficult and less attractive for those trained in the EU to migrate to the UK, we are instead seeing more professionals migrating from Asia and Africa. However, the top five source countries for international doctors over the last 10 years have remained the same, with increasing numbers of IMGs working in the UK from India, Pakistan, Nigeria, Egypt, and Bangladesh.^12^ Interestingly, all these nations are previous UK colonies, and three of these five countries are on the WHO red list. For many of these countries, cultural and historical ties through the Commonwealth, coupled with English being the primary language of medical education, make the UK a natural destination.

How do we justify our recruitment of these valuable healthcare professionals from countries that have a much greater need than our own? Provision of support and equitable training opportunities are key; however, this is unfortunately an area in which robust educational research is lacking.^14^ Clinical and educational supervisors have limited guidance as to how best to support international colleagues in UK training schemes, and this needs to be urgently addressed. Currently, postgraduate medical training in the UK also comes with significant levels of uncertainty and challenge for international doctors. A 2023 survey of IMGs in GP training suggested that around 50% had issues with the visa process^15^ making their roles here precarious and potentially short term. Failure to provide doctors with indefinite leave to remain is projected to result in the potential loss of over 1000 fully qualified GPs to the NHS. This has major impacts for our patients, but especially for the individuals themselves.

Brexit has changed health care in the UK in a number of ways^16^; however, our reliance on healthcare workers from abroad is not new and is likely to continue as we face a rapidly growing population and ever-increasing gaps in healthcare provision. Increasing diversity within the UK workforce should be expected to reflect the diversity within the general population. As healthcare professionals we are expected to work with the public and patients from a wide range of settings. Our international colleagues have cultural understanding and expertise that can help us in engaging patients from minority ethnic groups who are most affected by poor access and health inequality.

Box 1 **What is the WHO red list?**

- There are currently 55 countries on the WHO red list including Pakistan, Nigeria, and Bangladesh.
- Red-list countries face the most pressing workforce challenges; a low density of health professionals including doctors, nurses, and midwives means access to health care is difficult.
- Nine per cent of UK doctors are from countries on the WHO red list.

So why have a red list at all if we are keen to welcome international professionals into the UK regardless of their country of origin? Certainly, the presence of these countries on the red list does little to stem the migration tide because the underlying drivers of migration remain unaddressed. If policymakers wish to curb the exodus of health professionals from low- and middle-income countries, the focus perhaps needs to shift towards tackling the root causes that compel doctors to leave — challenges that often lie well beyond the scope of recruitment codes alone.

We would argue, however, that the red list is important; it reminds us that, despite the many workforce challenges we face in the UK, we still have access to excellent training and the benefits of living in a society with minimal personal risk. We have a moral responsibility to support our international colleagues who choose to migrate and offer them the world-class training opportunities that they seek. We must seek to understand how best to support our international colleagues and to challenge the inequality and discrimination they face.
